# Diffusibility Enhancement of Rejuvenator by Epoxidized Soybean Oil and Its Influence on the Performance of Recycled Hot Mix Asphalt Mixtures

**DOI:** 10.3390/ma11050833

**Published:** 2018-05-18

**Authors:** Dongliang Kuang, Yuan Jiao, Zhou Ye, Zaihong Lu, Huaxin Chen, Jianying Yu, Ning liu

**Affiliations:** 1 School of Materials Science and Engineering, Chang’an University, Xi’an 710064, China; kuangdl@163.com (D.K.); 15114863419@163.com (Y.J.); joe_yezhou@163.com (Z.Y.); chenhx_paper@163.com (H.C.); 2 Inner Mongolia Transportation Design Institute Co., Ltd., Huhhot 010000, China; luzaihongnm@163.com; 3 State Key Laboratory of Silicate Materials for Architectures, Wuhan University of Technology, Wuhan 430070, China; 4 Highway Researching and Designing Institute of Qinghai Province, Xining 810001, China; 1969ln@163.com

**Keywords:** reclaimed asphalt pavement, recycling, epoxidized soybean oil, rejuvenator, diffusing

## Abstract

Epoxidized soybean oil (ESO) was employed as a novel penetrant cooperating with a conventional rejuvenator (CR) for the recycling of reclaimed asphalt pavement (RAP). The influence of ESO on the diffusibility and the regenerating effects of CR on RAP were investigated. The diffusibility testing result shows that the diffusibility of CR is enhanced by the addition of ESO because the epoxy group in ESO can facilitate asphaltene dispersion due to its high polarity, which simultaneously reduces the viscosity and improves the fluidity of aged bitumen so as to allow diffusion of the rejuvenator into the aged bitumen. Road performance testing of a recycled hot mix asphalt mixture (RHMA) indicates that the fatigue and cracking resistance properties as well as the water stability of RHMA containing CR can be improved by the addition of ESO due to the diffusibility enhancement of CR, which boosts the regenerating effect of CR on aged bitumen in RAP. The fatigue and cracking resistance properties as well as the water stability of the recycled hot mix asphalt mixture containing CR with 7 wt % ESO approximate those of the hot mix asphalt mixture composed of the same virgin aggregates and bitumen. Taking into account the rutting resistance decline versus the addition of ESO, the content of ESO should not exceed 7 wt % of the conventional rejuvenator.

## 1. Introduction

In the recycling of reclaimed asphalt pavement (RAP), the RAP is blended with virgin aggregate, virgin bitumen as well as a rejuvenator to fabricate recycled hot mix asphalt (RHMA), which can conserve natural resources and reduce environmental pollution [1,2,3].

RAP usually has lower road performance due to the aging of bitumen [4,5,6]. A rejuvenator that can reconstitute the chemical component and colloidal structure of aged bitumen to regenerate its performance [7,8] is crucial for the recycling of RAP. In previous studies, it was reported that the rejuvenator and aged bitumen were initially blended evenly, and then a series of testing, including the physical properties, rheological properties and chemical components, was conducted on the blends to investigate the influence of the rejuvenator on aged bitumen; the results indicate that the aged bitumen can reach the target performance grade if the appropriate amount of the rejuvenator is determined and added [9,10,11,12].

However, for the practical recycling process of RHMA, the rejuvenator is first sprinkled on the surface of RAP and then blended with virgin aggregate as well as virgin bitumen [13]. The processing time usually lasts 45 to 60 s, so it is impossible for the rejuvenator to be uniformly mixed with aged bitumen in RAP as well as added virgin bitumen [14]. Therefore, the regeneration of aged bitumen in RAP is essentially dependent on the diffusion of the rejuvenator [15]. Previous research has revealed that the hardness and viscosity of aged bitumen in RAP are higher than unaged bitumen [16], which decreases the movement of the bitumen molecule; therefore, the fluidity of aged bitumen is poor, and the diffusion of the rejuvenator into aged bitumen is obstructed [17]. In order to enhance the diffusibility of the rejuvenator into aged bitumen, some attempts such as decreasing the viscosity of the rejuvenator and increasing the preheating temperature of RAP have been made [18]; nevertheless, the improvement was finite. Therefore, it is necessary to develop new methods to enhance the diffusibility of the rejuvenator.

As mentioned above, the high hardness and viscosity of the aged bitumen make the rejuvenator diffusion into aged bitumen difficult, which can be ascribed to the high asphaltene content [19]. Therefore, the diffusibility of the rejuvenator can be enhanced by simultaneously reducing the asphaltene content and by increasing the fluidity of the aged bitumen.

Epoxidized soybean oil (ESO), a kind of plasticizer, has been widely applied in the production of plastic, rubber and paint due to good fluidity, penetrability and temperature stability [20]. Recently, ESO has been used for the modification of oxidized bitumen; the results indicate that ESO displays good compatibility with the oxidized bitumen due to the polar epoxy group in ESO [21] and can facilitate asphaltene dispersion [22], which benefits the colloidal structure transformation of the oxidized bitumen from gel to sol-gel; therefore, the fluidity of oxidized bitumen can be enhanced and the viscosity can be reduced [23].

In this paper, ESO was adopted as a novel penetrant with a conventional rejuvenator (CR), and these were applied together for the recycling of RAP. In addition, the effects of ESO on the diffusibility and regenerating effects of the conventional rejuvenator were investigated.

## 2. Materials and Methods

### 2.1. Materials

Bitumen, i.e., AH-70 paving bitumen, was supplied by China Offshore Bitumen Co., Ltd., Taizhou, China. The physical properties and chemical components of the bitumen are listed in Table 1.

CR was prepared in the laboratory, and its physical properties are shown in Table 2.

ESO was supplied by Tongxiang Chemical Co., Ltd. In Tongxiang, China. The properties of ESO are listed in Table 3.

RAP was obtained from Jingzhu Expressway in Hubei Province, China. The bitumen and aggregates in RAP were separated according to ASTM D2172 [24]. The asphalt-aggregate ratio of RAP was 3.8 wt %, determined according to ASTM D6307 [25]. The properties and chemical components of the aged bitumen and the gradation of the aggregates in RAP are displayed by Table 4 and Table 5, respectively. Virgin aggregates, limestone coarse aggregate with a maximum sieve size of 19 mm and fine aggregate were supplied by Beishan Stone Factory in Chibi, China. 

### 2.2. Design and Preparation of RHMA

In this paper, AC-20 was selected as the objective gradation for RHMA. The Marshall Design procedure was used for the designing of RHMA of AC-20; the content of RAP was 30 wt %. The designed gradation is presented in Table 6. The optimum asphalt aggregate ratio of 4.2 wt % was determined according to Marshall testing.

Preparation of RHMA: first, RAP was heated to 110 °C; then, the rejuvenator plus ESO heated to 100 °C as well as virgin coarse aggregates heated to 180 °C were placed in a stirrer at 180 °C and blended for 10 s. Then, virgin bitumen heated to 150 °C was added and blended for 90 s; finally, mineral powder was added and continuously blended for 90 s.

For comparison, a virgin hot mix asphalt (VHMA) mixture of AC-20 composed of the same virgin bitumen and virgin aggregates as RHMA was also prepared as the control sample; the optimum asphalt aggregate ratio was 3.8 wt %. The selected gradation is presented in Table 7. The performance of VHMA is described in Table 8.

### 2.3. Diffusibility Testing Procedure of Rejuvenators

The diffusibility of the rejuvenators was tested according to the following procedure reported in previous research [26]:

- Aged bitumen heated to 140 °C was poured into penetration molds A and B, of which the inner diameter and height were 55 mm and 35 mm, respectively, and was then placed in an oven at a temperature of 110 °C for 30 min. Then, the rejuvenator heated to 110 °C was coated on the surface of the aged bitumen and maintained in an oven at 110 °C for 10 min to ensure the rejuvenator was uniformly scattered on the surface of the aged bitumen. The dosages of the aged bitumen and rejuvenator were 50 g and 5 g, respectively.
- Mold B was placed in an oven at a different temperature for a certain time or constant temperature for different times, and then continuously placed in an oven without power supply for 2.5–3 h, allowing the temperature to decrease to room temperature gradually to reduce the influence of interface contraction induced by a rapid decline in temperature on the flatness of the rejuvenator coat.

**Scheme 1:**
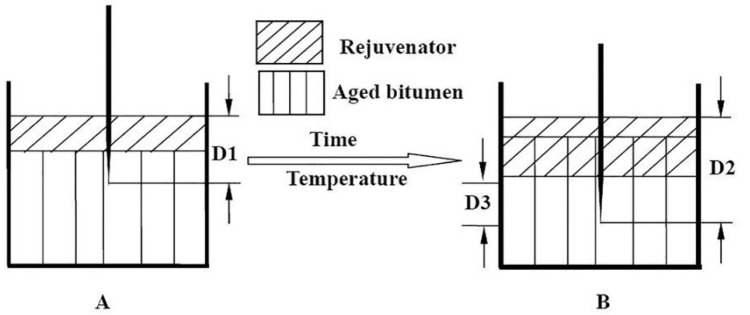
Synthesis of rejuvenator diffusion into aged bitumen by penetration testing.

As observed in Scheme 1, the penetrations of aged bitumen with the rejuvenator coat before and after the diffusion experiment were D1 and D2, respectively. The difference (D3) between D1 and D2 was used to characterize the diffusibility of the rejuvenator. The larger the D3 value, the better the diffusibility.

The temperature and time of the diffusing experiment were 160 °C and 4 h, respectively. The aged bitumen was produced by artificial aging in the laboratory. The weight of aged bitumen and rejuvenator were 50 g and 5 g, respectively, and the ESO dosages were 1 wt %, 3 wt %, 5 wt % and 7 wt % of the rejuvenator weight.

### 2.4. Performance Testing of Asphalt Mixtures

The rutting depth at 60 min (d_60_) and dynamic stability (DS) of the mixtures were tested according to ASTM D2172 [27]. The remnant stability (MS_0_) and freeze-thaw strength ratio (TSR) of the mixtures were tested according to ASTM D4867 [28] and ASTM D1559 [29], respectively.

The beam fatigue experiments of the samples at 15 °C were performed by the UTM dynamic servo hydraulic produced by IPC Co., Ltd., Sydney, Australia according to ASTM D32 [30]. The experiment was conducted under a stress-control model with a speed of 50 mm/min and frequency of 10 Hz; the loading time and unloading time were 0.1 s and 0.9 s, respectively; the testing stress was selected as 0.2 P, 0.3 P, 0.4 P and 0.5 P (P is the failure stress of each kind of asphalt mixture). 

Under the stress-control model, the fatigue properties can be depicted by the following fatigue equation.

(1) $$Ln\left(N_{f}\right)=-nLn\left(\sigma_{0}\right)+Ln\left(K\right)$$

where $N_{f}$ is the cycle number to failure; $\sigma_{0}$ is the loading stress; K and n are constants. After linear simulation, the constants K and n can be obtained. The fatigue properties of the mixture can be evaluated by constants K and n.

The bending test was employed to characterize the crack resistance of the asphalt mixture under a minus temperature temperature, which was also conducted by the UTM dynamic servo hydraulic produced by IPC Co., Ltd., Sydney, Australia, according to ASTM D32 [30]. The experiment was performed at −10 °C, and the loading rate was 50 mm/min.

## 3. Results and Discussion

### 3.1. Effect of ESO on the Diffusibility of CR

**Figure 1:**
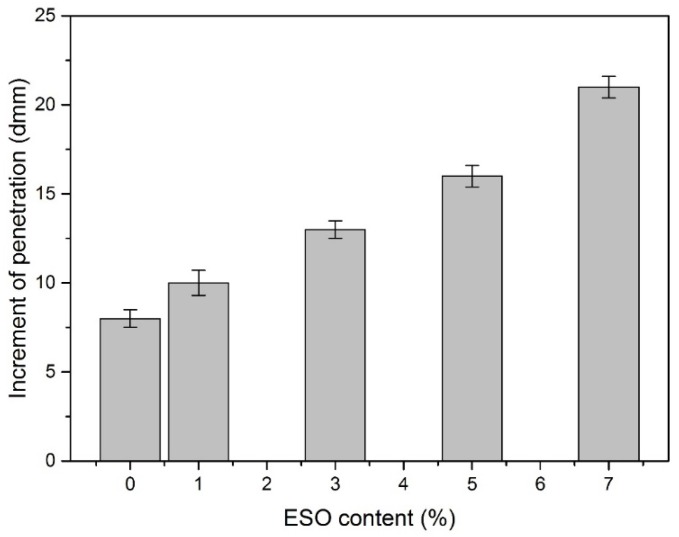
Penetration increment versus ESO.

Figure 1 reveals the penetration increment of aged bitumen covered by CR cooperating with ESO after being placed in an oven at 160 °C for 4 h. As observed from Figure 1, the penetration increment of aged bitumen covered by CR increased with the addition of ESO, which indicates the diffusibility of CR is enhanced by ESO. It can be explained as follows: the polar epoxy group included in ESO contributes to the asphaltene dispersion in the resin and aromatics, and then the viscosity of aged bitumen is reduced and the fluidity of aged bitumen is improved, which are helpful for the enhancement of diffusibility.

Compared with the penetration increment of aged bitumen with a CR coat of 8 dmm, the penetration increment of the aged bitumen with the CR coat cooperating with 7 wt % ESO reaches 21 dmm, which is 2.6 times the penetration increment of aged bitumen with the CR coat.

### 3.2. Effect of CR on the Performance of RHMA

Table 9 displays the performance of RHMA with different contents of CR. It can be found that both cycle number to failure and water stability of RHMA increased with an increase in the CR content; when the CR content approaches 20 wt %, the water stability can meet the application requirements. It can be also observed from the table that the DS of the RHAM with 15 wt % and 20% CR are 1220 time/mm and 920 time/mm, respectively, which are 46% and 35% of that of the RHMA without CR, indicating the rutting resistance of the RHMA is sharply reduced by a high content of the rejuvenator. In view of this fact, the CR content is selected as 10 wt % in the following experiment in this paper.

### 3.3. Effect of ESO on the Performance of RHMA with CR

#### 3.3.1. Fatigue Properties

**Figure 2:**
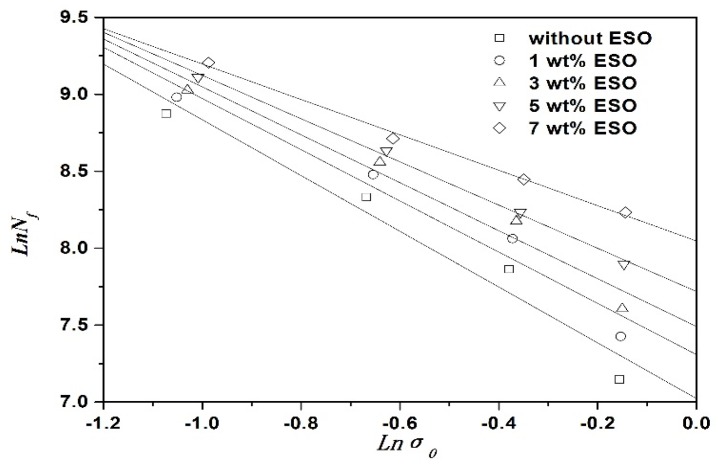
Fatigue curves of RHMA with CR incorporated with ESO.

The fatigue properties of RHMA with CR incorporated with different contents of ESO are shown in Figure 2 and Table 10. As displayed in Figure 2, the cycle number to failure of the RHMA with CR incorporated with ESO increases with the increase in ESO at different stress levels at 15 °C, indicating that the fatigue properties of the RHMA with CR can be improved by ESO. This can be explained as follows: CR containing ESO diffuses into aged bitumen more easily; therefore, the aged bitumen in RAP can be regenerated more effectively, which improves the fatigue properties of the RHMA.

Table 10 shows the parameters for the fatigue equation of RHMA with CR with different contents of ESO. It can be observed that the values of *K* and *n* of RHMA with CR increase with the addition of ESO, which also indicates that the RHMA containing the rejuvenator with ESO exhibits better fatigue properties [31]. The cycle number to failure of RHMA containing the CR with 7 wt % ESO is 9945, which nearly approaches the cycle number to failure of VHMA.

Furthermore, it can be also observed in Table 10 that the cycle number to failure of RHMA containing CR with 7 wt % ESO is 1.39 times that of RHMA containing CR without ESO under a 0.2 stress ratio; whereas, for the cycle number to failure of RHMA containing CR with 7 wt % ESO under the 0.5 stress ratio, it is 2.95 times that of RHMA containing CR without ESO under the 0.5 stress ratio, which indicates that ESO is more effective with respect to the fatigue property enhancement of RHMA containing CR under a high stress ratio.

#### 3.3.2. Cracking Resistance Properties

**Figure 3:**
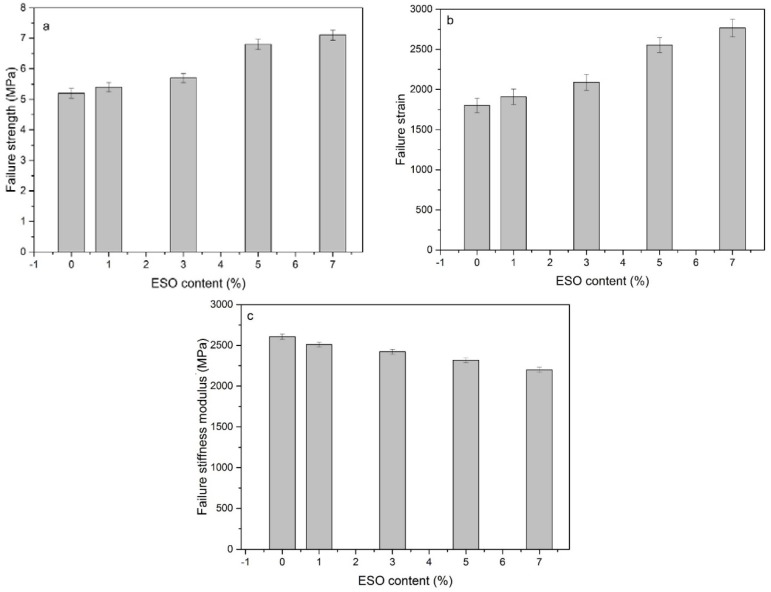
Beam bend results of RHMA with CR incorporated with ESO: (**a**) Failure strength; (**b**) Failure strain; (**c**) Failure stiffness modulus.

Figure 3 reveals the failure strength, failure strain and failure stiffness modulus of RHMA containing CR incorporated with different contents of ESO at −10 °C. It can be observed in Figure 3 that, as ESO increases, the failure strength and failure strain increase, whereas the failure stiffness modulus declines at the same time, indicating that the cracking resistance properties of RHMA with CR are improved by adding ESO. This can be ascribed to the chemical interaction between the epoxy groups which benefits the viscosity decline and molecule movement; therefore, the mixing between CR and aged bitumen as well as added virgin bitumen becomes more even. Consequently, the property restoration of aged bitumen is intensified, which significantly contributes to the enhancement of the cracking resistance of RHMA under a low temperature.

Furthermore, it can be also observed from Figure 3 that the failure strength and failure strain of RHMA containing CR with 7 wt % ESO can reach 7.3 MPa and 2767, respectively, i.e., 1.36 times and 1.54 times the failure strength and failure strain of RHMA containing only CR.

#### 3.3.3. Water Stability

**Figure 4:**
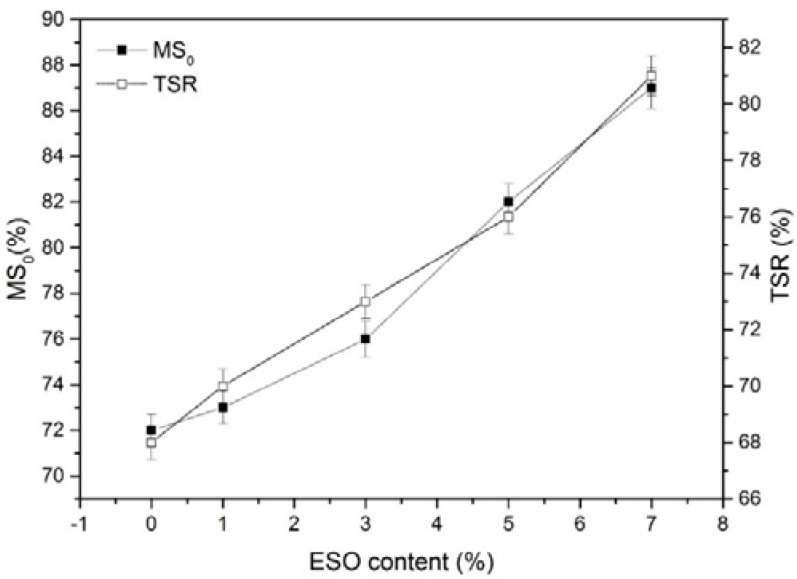
Water stability of RHMA with CR incorporated with ESO.

Figure 4 shows the MS_0_ and TSR of RHMA containing CR incorporated with different contents of ESO. It can be seen from Figure 4 that the MS_0_ and TSR of RHMA containing CR increase with the addition of ESO, indicating that the water stability of RHMA with CR is enhanced by ESO, which can be ascribed to the fact that ESO can facilitate CR diffusion into aged bitumen, wrapping around the surface of reclaimed aggregates by reducing the viscosity and increasing the fluidity of the aged bitumen, which benefits the compatibility between aged bitumen and CR as well as added virgin bitumen. Therefore the water stability of RHMA containing CR is improved by ESO. It can also be observed from Figure 4 that when the ESO content approaches 7 wt %, the MS_0_ and TSR of the RHMA containing CR can reach 87% and 81%, respectively, which is close to those of the VHMA.

#### 3.3.4. Rutting Resistance

**Figure 5:**
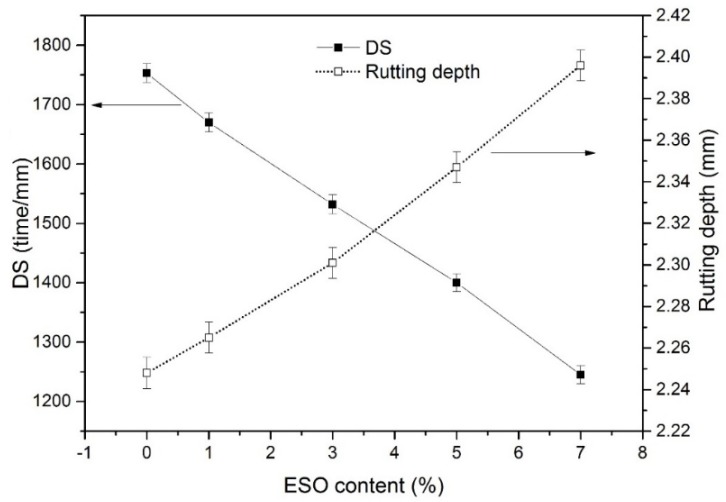
Rutting test result of RHMA with CR incorporated with ESO.

Figure 5 shows the DS and d_60_ of RHMA containing CR incorporated with different contents of ESO. It can be found that with an increase in ESO, the d_60_ increases, whereas DS declines, which indicates the rutting resistance of RHMA-containing rejuvenators is reduced by ESO. This can be explained as follows: ESO facilitates rejuvenator diffusion into aged bitumen and intensifies the mixing between aged bitumen and the rejuvenator as well as added virgin bitumen, which adversely affects the rutting resistance of the RHMA under high temperature; therefore, the d_60_ increases, whereas DS declines. However, when the ESO content is between 5 wt % and 7 wt %, the DS of the RHMA-containing rejuvenator can still satisfy the application requirements.

## 4. Conclusions

Epoxidized soybean oil was used as a penetrant to improve the diffusibility of a conventional rejuvenator and was applied for the recycling of reclaimed asphalt pavement together with a conventional rejuvenator. The influence of epoxidized soybean oil on the diffusibility of the conventional rejuvenator and the performance of a recycled hot mix asphalt mixture with the conventional rejuvenator were investigated. The conclusions were as follows:

1. The diffusibility of the conventional rejuvenator was significantly enhanced by ESO because the epoxy group included in ESO contributes to the asphaltene dispersion in resin and aromatics due to its high polarity. In addition, the fluidity of aged bitumen is improved and the viscosity is reduced, which are helpful for rejuvenator diffusion into aged bitumen.
2. The fatigue property, cracking resistance property as well as water stability of RHMA with the conventional rejuvenator were improved by ESO due to its facilitation of the diffusibility of the conventional rejuvenator. The fatigue property, cracking resistance property and water stability of the RHMA-containing conventional rejuvenator incorporated with 7 wt % approached those of the VHMA composed of the same virgin bitumen and virgin aggregates as RHMA.
3. The conventional rejuvenator with ESO improved the fatigue property, cracking resistance property as well as water stability of the RHMA at a lower content; thus, the negative effect of the high rejuvenator content on the rutting resistance of the RHMA was eliminated. Although the rutting properties of the RHMA-containing conventional rejuvenator incorporated with 7% ESO declined compared to RHMA with only the conventional rejuvenator, they still approached those of the VHMA and meet the application requirements.

## Figures and Tables

**Table 1:** Physical properties and chemical components of AH-70.

Items		Value
Physical properties	Softening point (°C)	47.2
	Ductility (15 °C, cm)	>150
	Penetration (25 °C, 0.1 mm)	70
	Viscosity (60 °C, Pa·s)	232
Chemical components	Saturates (%)	12.2
	Aromatics (%)	50.2
	Resins (%)	26.1
	Asphaltenes (%)	11.5

**Table 2:** Properties of CR.

Physical Properties	Items
Viscosity at 60 °C (cst)	1700
Flash point (°C)	>220
Viscosity ratio before and after TFOT	2.5
Weight loss after TFOT (%)	−2.6
Saturates content (%)	<30

**Table 3:** Properties of ESO.

Properties	Value
Molecular	1000
Viscosity (cps, 25 °C)	350
Acid value	0.45
Iodine value (%)	4.0
Flash point (°C)	280
Epoxy value (%)	6.1
Weight loss after heating (%)	0.08
Specific gravity (25 °C)	0.982

**Table 4:** Physical properties and chemical components of aged bitumen.

Items		Value
Physical properties	Softening point (°C)	58.3
	Ductility (15 °C, cm)	9.6
	Penetration (25 °C, 0.1 mm)	30
	Viscosity (60 °C, Pa·s)	410
Chemical components	Saturates (%)	11.2
	Aromatics (%)	31.3
	Resins (%)	29.1
	Asphaltenes (%)	28.4

**Table 5:** Gradation of aggregates in RAP.

Sieve (mm)	19	16	13.2	9.5	4.75	2.36	1.18	0.6	0.3	0.15	0.075
Passing (%)	100	99.2	98.3	90.9	68.4	50.3	34.1	26	17.4	14.3	4.7

**Table 6:** Gradation of RHMA.

Sieve (mm)	19	16	13.2	9.5	4.75	2.36	1.18	0.6	0.3	0.15	0.075
Passing (%)	96.7	87.5	78.3	60.2	42.2	25.3	18.7	15.4	11.5	9.2	6.1

**Table 7:** Gradation of VHMA.

Sieve (mm)	19	16	13.2	9.5	4.75	2.36	1.18	0.6	0.3	0.15	0.075
Passing (%)	97.1	87.5	76.6	55.7	38.2	27.5	18.8	16.2	12.7	10.2	5.1

**Table 8:** Performance of VHMA.

Performance		VHMA
Rutting resistance	d_60_ (mm)	2.263
	DS (time/mm)	1300
Water stability	MS_0_ (%)	89
	TSR (%)	83
Fatigue property	N_f_ at 0.2 P (times)	10,350
	N_f_ at 0.3 P (times)	7951
	N_f_ at 0.4 P (times)	4642
	N_f_ at 0.5 P (times)	3617
Crack resistance	Failure strength (MPa)	7.3
	Failure strain (%)	2990
	Failure stiffness modulus (MPa)	2120

**Table 9:** Performance of RHMA with different CR contents.

Performance		CR Content (%)				
		0	5	10	15	20
Rutting resistance	d_60_ (mm)	2.011	2.137	2.248	2.352	2.473
	DS (time/mm)	2650	2271	1753	1220	920
Water stability	MS_0_ (%)	67	70	72	78	82
	TSR (%)	61	64	68	73	79
Fatigue property	N_f_ at 0.2 P (times)	5860	6760	7145	8542	9350
	N_f_ at 0.3 P (times)	3155	3555	4153	6340	7620
	N_f_ at 0.4 P (times)	1375	2100	2600	3650	4100
	N_f_ at 0.5 P (times)	650	1000	1270	2005	2410
Crack resistance	Failure strength (MPa)	4.2	4.5	5.2	5.8	6.7
	Failure strain (%)	1400	1550	1800	2072	2300
	Failure stiffness modulus (MPa)	3020	2800	2605	2317	2150

**Table 10:** Parameters for the fatigue equation of RHMA with CR incorporated with ESO.

ESO Content (%)	*N_f_*				Fatigue Equation	Equation Parameters	
	Stress Ratio					*K*	*n*
	0.2	0.3	0.4	0.5			
0	7145	4153	2600	1270	$LnN_{f}=-1.149Ln\sigma_{0}+7.022$	1121	1.149
1	7948	4810	3170	1680	$LnN_{f}=-1.405Ln\sigma_{0}+7.308$	1492	1.405
3	8327	5210	3550	2010	$LnN_{f}=-1.577Ln\sigma_{0}+7.491$	1791	1.577
5	9054	5620	3770	2690	$LnN_{f}=-1.665Ln\sigma_{0}+7.718$	2248	1.665
7	9945	6074	4660	3757	$LnN_{f}=-1.812Ln\sigma_{0}+8.047$	3124	1.812
